# Diagnostic Performance and Confidence Calibration of Large Language Models for Bone Tumor Radiographs

**DOI:** 10.3390/diagnostics16101460

**Published:** 2026-05-11

**Authors:** Sanjana Arun, Eujung Park, Katja Klosterman, Carissa Zhu, Ronak Arun, Palmer Wrigley Stratton, Hamsa Gangaswamiah

**Affiliations:** 1 College of Medicine Phoenix, University of Arizona, Phoenix, AZ 85004, USA; eujungpark@arizona.edu (E.P.); katjakloste@arizona.edu (K.K.); carissazhu@arizona.edu (C.Z.); 2 Language School and Arts Department, University of Michigan, Ann Arbor, MI 48109, USA; ronakarun2004@gmail.com; 3 Biological Sciences, Arizona State University, Tempe, AZ 85287, USA; pswrigle@asu.edu; 4 Robert Wood Johnson Barnabas Health, New Brunswick, NJ 08901, USA; hamsa_jee@yahoo.com

**Keywords:** artificial intelligence, radiographs, large language models, bone tumors, diagnostic accuracy, medical imaging

## Abstract

**Background/Objectives:** Large language models (LLMs) are increasingly applied to medical image interpretation; however, their diagnostic accuracy and reliability in musculoskeletal radiology remain uncertain. This study evaluates the diagnostic performance and confidence calibration of LLMs in detecting and classifying bone tumors on radiographs. **Methods:** This retrospective observational study analyzed a dataset of 257 radiographs with confirmed diagnoses obtained from Radiopaedia, including normal studies and a spectrum of benign and malignant bone tumors. Cases were selected to ensure representation across multiple tumor types. Three LLMs (ChatGPT 5.3, X-ray Interpreter GPT-4.1, and X-ray Interpreter Gemini) evaluated each image using a standardized prompt assessing abnormality detection, tumor detection, classification, and confidence. Outcomes included diagnostic accuracy, false positive abnormality rates, false negative rates, tumor hallucination rates, and confidence calibration. **Results:** Abnormality detection was high across models, with Gemini demonstrating the highest sensitivity (up to 100%). Tumor detection was strongest in lesions with characteristic features, including osteosarcoma and osteochondroma. False negative rates varied substantially, with GPT-4.1 demonstrating the highest rate (29.9%), followed by ChatGPT (24.8%) and Gemini (6.6%). Primary diagnostic accuracy was highest for osteosarcoma in GPT-4.1 (80%), while ChatGPT 5.3 performed best in benign lesions, including osteochondroma (84.6%) and non-ossifying fibroma (76.9%). Tumor subtype classification remained limited across all models and was poorest for Ewing sarcoma (0% in ChatGPT and GPT-4.1; 10.3% in Gemini). False positive abnormality rates were highest in GPT-4.1 (40.7%), followed by Gemini (25.9%) and ChatGPT (13.5%). Tumor hallucination occurred only in Gemini (12.3%). All models demonstrated confidence miscalibration, with higher confidence observed in incorrect predictions and in tumor-negative cases. **Conclusions:** LLMs demonstrate strong performance in detecting radiographic abnormalities but remain limited in tumor subtype classification, particularly for diagnostically challenging lesions such as Ewing sarcoma. Elevated false positive and false negative rates, along with systematic overconfidence—especially in GPT-4.1—highlight important limitations for clinical use. These findings support the role of LLMs as adjunctive tools rather than independent diagnostic systems.

## 1. Introduction

The application of artificial intelligence (AI) in medical imaging has expanded rapidly, with increasing interest in tools that can assist in radiographic interpretation and clinical decision-making. Recent advances in large language models (LLMs), including ChatGPT and Gemini, have introduced multimodal capabilities that allow for simultaneous processing of textual and visual data. These systems have shown promise in radiology workflows, where they may assist with diagnostic reasoning and differential diagnosis generation. However, their performance in musculoskeletal radiology remains incompletely characterized.

Although commonly referred to as large language models (LLMs), the systems evaluated in this study incorporate multimodal capabilities that enable image interpretation and can be more precisely categorized as vision-language models (VLMs). In this study, we use the term LLM to reflect their real-world deployment as publicly accessible AI tools.

Bone tumor diagnosis represents a particularly complex clinical task requiring integration of imaging, clinical context, and pathology. Plain radiography remains the initial and most widely used imaging modality for evaluating suspected bone lesions, allowing characterization of lesion location, margins, and matrix features [1]. In many cases, radiographs can narrow the differential diagnosis; however, overlap between benign and malignant lesions limits definitive interpretation [2]. As a result, histopathologic evaluation via biopsy remains the gold standard for diagnosis, particularly in lesions with aggressive or indeterminate features [3,4]. This distinction highlights that radiographs primarily serve a role in detection and characterization, whereas definitive diagnosis requires tissue confirmation.

Recent studies have begun to evaluate the performance of LLMs in musculoskeletal radiology. In a comparative study of ChatGPT and radiologists, GPT-4-based models demonstrated diagnostic performance comparable to radiology residents but remained inferior to board-certified radiologists, particularly when interpreting imaging directly rather than textual descriptions [5]. Similarly, large-scale analyses of bone tumor diagnosis have shown that while ChatGPT may improve efficiency and reduce missed diagnoses, it struggles with complex cases due to overlapping imaging features and diagnostic ambiguity [6]. Additional work integrating ChatGPT with deep learning models has demonstrated potential improvements in diagnostic workflows, particularly in oncologic imaging; however, these systems remain dependent on underlying model accuracy and may propagate errors [7]. These findings suggest that although LLMs may assist in narrowing differential diagnoses, their reliability in tumor classification remains limited.

A critical limitation of LLMs is confidence miscalibration, in which incorrect predictions are assigned high confidence. This phenomenon poses significant risks in clinical settings, where overconfident errors may influence diagnostic decision-making. In addition, LLMs may exhibit hallucination, defined as the generation of plausible but incorrect outputs. In radiographic interpretation, this may manifest as the identification of pathology, including tumors, in normal images. While hallucination has been widely described in natural language applications, its impact in medical imaging remains an area of active investigation.

Given these limitations, further evaluation of LLM performance across a range of musculoskeletal pathologies is needed. In particular, bone tumors provide a useful test case due to their diagnostic complexity and reliance on imaging interpretation. In this study, we evaluate the diagnostic performance and confidence calibration of multiple LLMs in the interpretation of bone tumor radiographs using a dataset of images with confirmed diagnoses. We assess abnormality detection, tumor identification, subtype classification, differential diagnosis generation, and confidence. We hypothesize that while LLMs will demonstrate high sensitivity for detecting abnormalities, they will show reduced accuracy in tumor subtype classification and exhibit significant confidence miscalibration.

## 2. Methods

### 2.1. Study Design and Setting

This study was a retrospective, observational analysis evaluating the diagnostic performance and confidence calibration of large language models (LLMs) in bone tumor radiograph interpretation. A curated dataset of radiographs with confirmed diagnoses was obtained from Radiopaedia, a peer-reviewed, open-edit educational radiology resource compiled by radiologists and radiology trainees worldwide. Radiopaedia provides publicly accessible cases with expert-reviewed interpretations, supporting its use as a reliable reference dataset. All images were used in accordance with Radiopaedia attribution guidelines. All images were de-identified and analyzed in a non-clinical research setting.

Radiographic images were obtained from Radiopaedia (https://radiopaedia.org), a peer-reviewed, open-access radiology resource curated by radiologists and radiology trainees worldwide [8]. Cases were selected across multiple tumor categories, including both benign and malignant lesions, to ensure a representative dataset of bone pathology. Only cases with confirmed diagnoses and sufficient image quality for interpretation were included. Images containing annotations, arrows, or overlays were excluded to minimize bias and ensure that model predictions were based solely on radiographic features.

For each included case, the radiograph was extracted and paired with its corresponding ground truth diagnosis as provided by the source database. To ensure proper attribution and reproducibility, the following metadata were recorded for each case: Radiopaedia identification number (rID), case contributor name, and source URL. A complete list of all included cases and associated attribution details is provided in Supplementary Table S1. All images were used in accordance with Radiopaedia attribution guidelines.

### 2.2. Data Processing and Analysis

A total of 257 radiographs (141 cases) were included. All analyses were performed at the level of individual radiographic images. When multiple images were available for a single case, each image was evaluated independently. Each case was assigned to a member of the study team, who uploaded the radiograph into one of three publicly accessible AI platforms: ChatGPT 5.3, X-ray Interpreter 4.1, or X-ray Interpreter Gemini. These platforms represent multimodal AI systems that integrate visual processing with language-based reasoning. All models were accessed via publicly available web-based interfaces and were not run locally or through application programming interfaces (APIs), in order to reflect real-world usage conditions. Radiographic images were downloaded from Radiopaedia and manually uploaded into each AI platform through its user interface. A standardized prompt was copied and applied consistently for each image. Each evaluation was performed in a new, independent session to minimize carryover effects. To ensure standardized evaluation, each team member used an identical prompt and recorded outputs in a shared spreadsheet (Microsoft Excel).

No clinical history, demographic information, or additional context was provided to the models. To minimize bias and prevent carryover effects, each case was evaluated in a new, independent session.

The standardized prompt was as follows:

“Please analyze the following radiograph and answer the questions below:

1. Does this radiograph appear normal or abnormal?
2. Is there evidence on this radiograph that suggests a bone tumor or bone malignancy? (Yes/No)
3. If a bone tumor is suspected, what is the most likely diagnosis?
4. Please provide the top three most likely diagnoses in order of likelihood.
5. How confident are you in your primary diagnosis? (0–100%)”

Model outputs were recorded and categorized. Abnormality detection and tumor detection were analyzed as distinct outcomes. Performance metrics included abnormality detection, tumor detection, primary diagnostic accuracy, and differential diagnosis inclusion (top three). False positive abnormality rates (normal radiographs classified as abnormal) and tumor hallucination rates (incorrect identification of tumor in normal radiographs) were calculated.

Descriptive statistics were used to summarize model performance, with categorical variables reported as proportions and percentages. Diagnostic accuracy was further evaluated within tumor-positive cases to assess model performance in identifying correct tumor subtypes. Additional performance metrics included abnormality detection rate, tumor detection rate, primary diagnostic accuracy, differential inclusion (top three), false positive abnormality rate, tumor hallucination rate, and mean confidence.

Comparative analyses between models were performed using chi-square tests to assess differences in categorical outcomes, including abnormality detection, tumor detection, and primary diagnostic accuracy. Pairwise comparisons between models were conducted where applicable. A *p*-value of <0.05 was considered statistically significant. Chi-square testing was selected due to the categorical nature of the outcome variables.

Confidence values were analyzed as continuous variables and summarized as mean percentages across all cases and within tumor-specific subgroups to evaluate model calibration. Conditional accuracy was also calculated as the proportion of correctly classified tumor subtypes among cases in which a tumor was detected.

All statistical analyses were conducted using Microsoft Excel (Microsoft Corp., Redmond, WA, USA).

### 2.3. Inclusion and Exclusion Criteria

Radiographs were included only if they had a confirmed diagnosis, as provided by the source database and verified through radiologist-reviewed case interpretations. Radiographs were included only if they had a confirmed diagnosis, as explicitly documented in the source database. Diagnoses in Radiopaedia are peer-reviewed and typically established through radiologist interpretation with supporting clinical, imaging, and/or histopathologic confirmation where available. Cases without clearly defined final diagnoses were excluded. Additional inclusion criteria were adequate image quality and representation of predefined diagnostic categories (normal, osteosarcoma, Ewing sarcoma, enchondroma, osteochondroma, non-ossifying fibroma, chondrosarcoma, and simple bone cyst).

Radiographs were excluded if the diagnosis was uncertain or not explicitly confirmed, if image quality was insufficient for interpretation, or if identifying patient information was present. Cases with significant confounding features, such as pathological fractures or extensive secondary changes, were excluded where these findings could bias model interpretation beyond intrinsic tumor characteristics.

### 2.4. Ethical Approval and Data Privacy

This study utilized publicly available, de-identified radiographic images and did not involve human subjects or direct patient interaction. As such, institutional review board approval was not required. All data were accessed in accordance with the source platform’s terms of use, and no protected health information was collected or stored.

### 2.5. Figure Generation

Figures were created using BioRender.

## 3. Results

**Figure 1:**
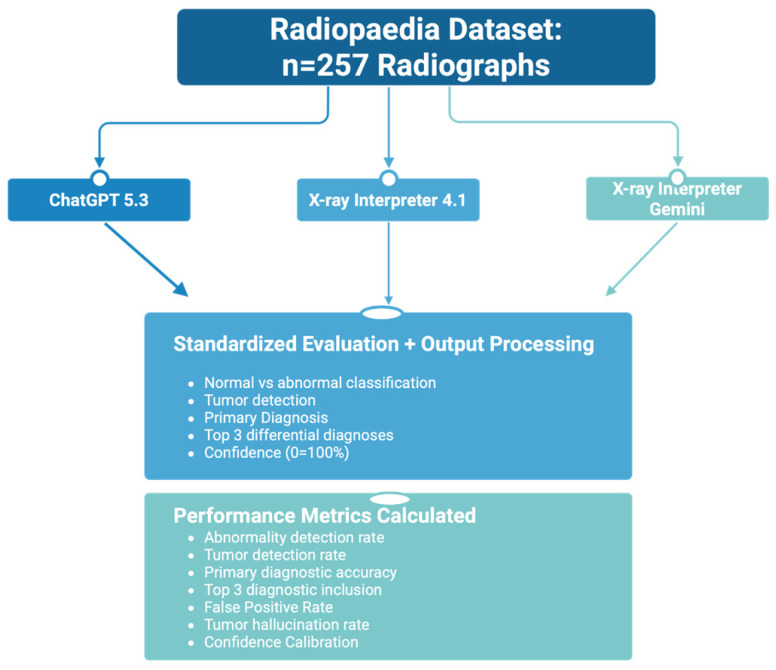
Study design and evaluation workflow. Overview of the study methodology for evaluating artificial intelligence (AI) models in bone tumor diagnosis using radiographic images. Radiographs representing seven tumor categories were obtained from Radiopaedia and input into three AI models (ChatGPT 5.3, X-ray Interpreter 4.1, and X-ray Interpreter Gemini). A standardized prompt was applied to each image, requiring classification of normal versus abnormal findings, assessment of tumor presence, identification of the most likely diagnosis, generation of a ranked differential diagnosis (top three), and assignment of diagnostic confidence. Model outputs were systematically recorded and used to calculate performance metrics, including abnormality detection rate, tumor detection rate, primary diagnostic accuracy, top-3 diagnostic accuracy, and confidence scores.

A total of 257 radiographs representing 141 unique cases were included in the analysis, spanning normal studies and a spectrum of benign and malignant bone tumors (Table 1, Figure 1). The dataset included osteosarcoma (*n* = 30), Ewing sarcoma (*n* = 29), chondrosarcoma (*n* = 25), enchondroma (*n* = 25), osteochondroma (*n* = 26), non-ossifying fibroma (*n* = 26), and simple bone cyst (*n* = 15), allowing for evaluation across lesions with varying radiographic complexity.

**Figure 2:**
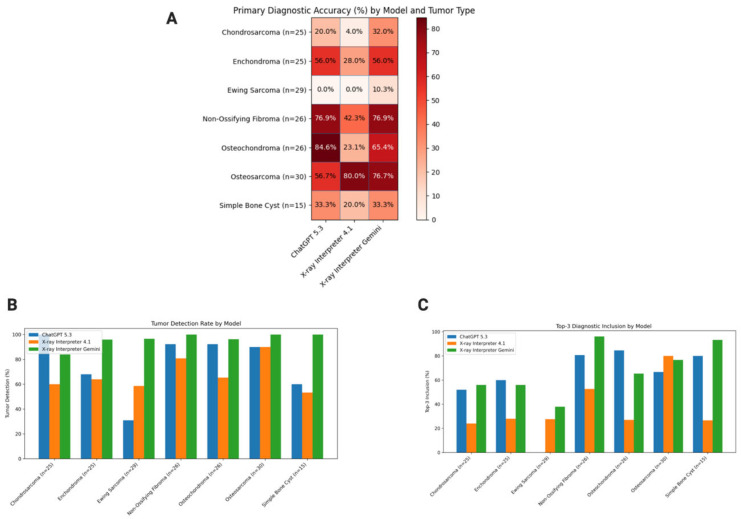
(**A**) Primary diagnostic accuracy of AI models across bone tumor types. Heat map illustrating the primary diagnostic accuracy (%) of three AI models across seven bone tumor categories. Each cell represents the proportion of cases in which the model’s top-ranked diagnosis matched the ground truth. Tumor types include osteosarcoma (*n* = 30), Ewing sarcoma (*n* = 29), chondrosarcoma (*n* = 25), enchondroma (*n* = 25), osteochondroma (*n* = 26), non-ossifying fibroma (*n* = 26), and simple bone cyst (*n* = 15). Darker red shading indicates higher diagnostic accuracy. Percentage values are displayed within each cell, with white text used for higher values to enhance contrast. Gridlines are included to improve readability and facilitate comparison across models and tumor types. (**B**) Tumor detection rate by AI model and tumor type. Grouped bar chart showing tumor detection rates (%), defined as the proportion of cases in which each model correctly identified the presence of a bone tumor. Performance is stratified by tumor type and compared across models, allowing direct visualization of detection capability across different pathological entities. (**C**) Top-3 diagnostic inclusion by AI model and tumor type. Grouped bar chart illustrating top-3 diagnostic inclusion (%), defined as the proportion of cases in which the correct diagnosis appeared within the model’s differential diagnosis. This metric reflects the ability of each model to consider the correct diagnosis even when not ranked as the primary prediction.

Across all cases, model performance varied substantially by task (Table 2, Figure 2). X-ray Interpreter Gemini demonstrated the highest abnormality detection (97.8%) and tumor detection rates (93.4%), significantly outperforming both ChatGPT (88.7% and 75.2%) and X-ray Interpreter 4.1 (82.4% and 70.1%). False negative rates, defined as the proportion of tumor-containing radiographs incorrectly classified as non-neoplastic, varied substantially across models (Table 2). GPT-4.1 demonstrated the highest false negative rate (29.9%), followed by ChatGPT (24.8%), while Gemini showed the lowest rate (6.6%), reflecting superior sensitivity for tumor detection. These differences were statistically significant for abnormality detection across all pairwise comparisons (*p* ≤ 0.02) and for tumor detection when comparing Gemini to both ChatGPT and GPT-4.1 (*p* < 0.001), while no significant difference was observed between ChatGPT and GPT-4.1 (*p* = 0.29) (Table 3 pairwise).

Primary diagnostic accuracy followed a similar trend, with Gemini achieving the highest overall accuracy (56.1%), followed by ChatGPT (52.6%) and GPT-4.1 (36.2%) (Figure 2A). GPT-4.1 performed significantly worse than both ChatGPT and Gemini (*p* < 0.001), whereas no significant difference was observed between ChatGPT and Gemini (*p* = 0.48). Despite comparable detection performance, GPT-4.1 consistently lagged in correct tumor classification (Table 3 Pairwise).

**Table 3:** Pairwise. Overall diagnostic performance of large language models in radiograph interpretation. Metrics are reported as weighted percentages across all diagnostic categories. Abnormality detection and tumor detection were analyzed as separate outcomes. False positive abnormality rates represent normal radiographs incorrectly classified as abnormal, while tumor hallucination refers to incorrect identification of tumor in normal images. False negative rate represents the proportion of tumor-containing radiographs incorrectly classified as non-neoplastic and was calculated as the complement of tumor detection rate. Mean confidence reflects the weighted average confidence % of the AI modality across all cases. Confidence (Tumor Present) and Confidence (No Tumor) represent mean confidence stratified by tumor detection status. Pairwise comparisons between models were performed using chi-square testing. Statistically significant differences were observed across abnormality detection, tumor detection, and primary diagnostic accuracy, with Gemini demonstrating significantly higher detection rates, while GPT-4.1 showed significantly lower diagnostic accuracy compared to other models.

Metric	Comparison	*p*-Value
**Abnormality Detection**	ChatGPT vs. GPT-4.1	0.02
**Abnormality Detection**	ChatGPT vs. Gemini	<0.001
**Abnormality Detection**	GPT-4.1 vs. Gemini	<0.001
**Tumor Detection**	ChatGPT vs. GPT-4.1	0.29
**Tumor Detection**	ChatGPT vs. Gemini	<0.001
**Tumor Detection**	GPT-4.1 vs. Gemini	<0.001
**Primary Diagnostic Accuracy**	ChatGPT vs. GPT-4.1	<0.001
**Primary Diagnostic Accuracy**	ChatGPT vs. Gemini	0.48
**Primary Diagnostic Accuracy**	GPT-4.1 vs. Gemini	<0.001

Performance varied considerably across tumor types (Table 4, Figure 2). Detection rates were highest for morphologically distinct lesions, including osteosarcoma and non-ossifying fibroma, where all models achieved ≥90% detection, with Gemini reaching 100% in both categories. In contrast, detection of Ewing sarcoma was markedly lower for ChatGPT (31.0%) and GPT-4.1 (58.6%), while Gemini maintained high sensitivity (96.6%), highlighting improved recognition of aggressive pathology.

Primary diagnostic accuracy demonstrated even greater variability. High accuracy was observed in osteochondroma (ChatGPT: 84.6%) and non-ossifying fibroma (ChatGPT and Gemini: 76.9%), whereas all models performed poorly in Ewing sarcoma classification, with 0% accuracy for both ChatGPT and GPT-4.1 and only 10.3% for Gemini (Figure 2A). Chondrosarcoma and simple bone cyst also showed relatively low primary accuracy across models, suggesting difficulty in distinguishing tumors with overlapping radiographic features.

Inclusion of the correct diagnosis within the top three differential diagnoses improved performance across all models (Figure 2C, Table 4). For example, Ewing sarcoma, which was not identified as the primary diagnosis by any model, was included in the top 3 differentials in 27.6% of GPT-4.1 cases and 37.9% of Gemini cases. Similarly, simple bone cyst inclusion reached 80.0% for ChatGPT and 93.3% for Gemini despite low primary accuracy, indicating that models often considered the correct diagnosis even when not ranking it first.

Conditional accuracy was consistently higher than overall primary accuracy across tumor types (Table 4, Figure 2), where conditional accuracy was defined as the proportion of correctly classified tumor subtypes among cases in which a tumor was correctly identified. This pattern was particularly evident for enchondroma (ChatGPT: 82.0%) and osteochondroma (ChatGPT: 91.7%), suggesting that once tumor presence was correctly identified, models demonstrated improved subtype classification.

False positive abnormality rates differed notably across models (Table 2). False positive abnormality was defined as the incorrect classification of a normal radiograph as abnormal. GPT-4.1 demonstrated the highest false positive rate (40.7%), compared to Gemini (25.9%) and ChatGPT (13.5%), indicating reduced specificity in normal radiographs. Tumor hallucination was not observed in ChatGPT or GPT-4.1 but occurred in 12.3% of Gemini interpretations, reflecting a tendency to overcall neoplastic processes in normal images.

**Figure 3:**
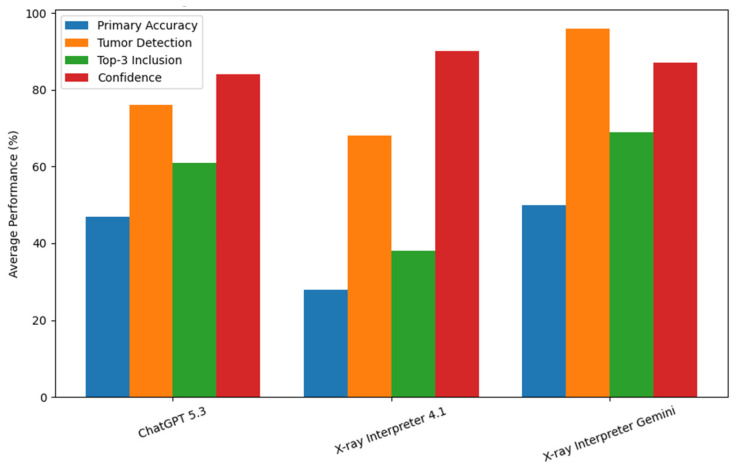
Overall performance and confidence comparison of AI models in bone tumor diagnosis. Grouped bar chart illustrating the average performance of each AI model across all tumor types. Metrics include primary diagnostic accuracy (%), tumor detection rate (%), top-3 diagnostic inclusion (%), and mean diagnostic confidence (%). Confidence values were derived from model-reported confidence scores averaged across tumor subgroups. Comparison of confidence and accuracy highlights differences in model calibration, with certain models demonstrating disproportionately high confidence relative to diagnostic accuracy.

Confidence analysis revealed important differences in model calibration (Figure 3). Confidence was defined as the model-reported percentage representing its certainty in the primary diagnosis. GPT-4.1 demonstrated the highest mean confidence (91.1%) despite the lowest diagnostic accuracy (36.2%), indicating a pattern of overconfidence. In contrast, ChatGPT exhibited lower mean confidence (85.4%) with higher diagnostic accuracy (52.6%), suggesting more appropriate calibration. Gemini demonstrated both high diagnostic performance (56.1%) and moderately high confidence (88.6%), reflecting comparatively balanced performance.

When stratified by tumor detection status, all models demonstrated higher confidence in cases where tumors were not identified compared to tumor-positive cases. This discrepancy was most pronounced in GPT-4.1, where mean confidence was 96.0% in tumor-negative cases compared to 84.0% in tumor-positive cases. ChatGPT and Gemini demonstrated smaller but consistent differences, with confidence values of 91.5% versus 83.5% and 92.5% versus 87.5%, respectively (Table 2).

At the tumor level, multiple subgroups demonstrated elevated confidence in incorrect predictions, particularly for GPT-4.1. For example, in Ewing sarcoma and enchondroma, incorrect classifications were associated with higher confidence than correct diagnoses, indicating systematic miscalibration (Table 2). Collectively, these findings suggest that while all models demonstrate high confidence, GPT-4.1 is most prone to overconfidence relative to its diagnostic accuracy, raising concerns regarding reliability in clinical application.

## 4. Discussion

This study demonstrates that large language models (LLMs) exhibit strong performance in detecting radiographic abnormalities and identifying the presence of bone tumors, but remain limited in accurate tumor subtype classification. Across all models, detection consistently outperformed diagnostic accuracy, highlighting the distinction between recognizing abnormal pathology and correctly characterizing tumor subtype. This finding is consistent with prior work demonstrating that conventional radiography is effective for initial lesion detection but has limited specificity for definitive diagnosis without additional imaging or histopathologic confirmation [1,2]. In clinical practice, accurate characterization of bone lesions often requires correlation with advanced imaging and biopsy, particularly for lesions with overlapping radiographic features [3,4].

Among the evaluated models, X-ray Interpreter Gemini demonstrated the highest overall performance, particularly in abnormality and tumor detection. Its strong performance likely reflects the ability of deep learning systems to identify visual patterns associated with pathology, a phenomenon well described in radiologic AI literature [9,10]. However, despite high sensitivity, primary diagnostic accuracy remained modest across all models. This reinforces the well-established limitation that radiographic appearance alone is frequently insufficient for precise tumor classification, particularly in lesions with nonspecific or heterogeneous features. Prior studies in musculoskeletal imaging have similarly shown that even experienced clinicians demonstrate variability in diagnostic accuracy when relying solely on plain radiographs [2,11].

In contrast, X-ray Interpreter 4.1 demonstrated the lowest diagnostic accuracy despite relatively preserved detection performance. This discrepancy was further accentuated by its consistently high confidence, indicating significant miscalibration. The presence of high confidence in incorrect predictions is particularly concerning in clinical contexts, as it may increase the risk of diagnostic error and inappropriate management. Prior work evaluating AI systems in medical imaging has highlighted that model confidence is often poorly calibrated and may not reliably reflect true predictive accuracy [12,13]. This phenomenon of overconfidence has been described as a key barrier to safe clinical deployment of AI systems, particularly in high-stakes diagnostic environments.

ChatGPT demonstrated intermediate performance, with more balanced diagnostic accuracy and confidence compared to GPT-4.1. While not the highest-performing model, its relatively better alignment between confidence and accuracy suggests improved calibration. This aligns with emerging literature suggesting that large language models may be more useful as decision-support tools that assist with reasoning and differential diagnosis rather than as primary diagnostic systems [5,6,7,14].

Tumor-specific analysis further highlights variability in model performance. All models performed poorly in Ewing sarcoma classification despite moderate to high detection rates, suggesting difficulty in identifying tumors with less distinct radiographic features. In contrast, benign or morphologically characteristic lesions, such as osteochondroma and non-ossifying fibroma, were more accurately classified. This pattern is consistent with established radiologic principles, where lesions with characteristic imaging features are more readily diagnosed, whereas others require histopathologic confirmation [3,4].

Importantly, top-3 diagnostic inclusion improved performance across all models, indicating that correct diagnoses were frequently present within the differential even when not ranked first. This suggests that LLMs may have value as adjunctive tools to assist in generating differential diagnoses rather than serving as standalone diagnostic systems. Similar findings have been reported in studies evaluating AI-assisted diagnostic reasoning, where models contribute meaningfully to hypothesis generation even when primary predictions are incorrect [14,15].

A key contribution of this study is the evaluation of model calibration. Across multiple tumor subgroups, particularly in GPT-4.1, incorrect predictions were often associated with equal or higher confidence than correct diagnoses, demonstrating systematic overconfidence. Notably, all models demonstrated higher confidence in cases in which tumors were not detected compared to tumor-positive cases, indicating a tendency toward overconfidence in clinically high-risk scenarios involving missed pathology. This finding has important implications for clinical safety, as overconfident incorrect outputs may reduce clinician skepticism and delay appropriate follow-up. Addressing calibration and uncertainty estimation has been identified as a critical step in the safe integration of AI into clinical workflows [12,13].

## 5. Limitations

This study has several limitations. First, the dataset was derived from publicly available radiographs, which may introduce bias. Because these images are widely accessible online, it is possible that models have been previously exposed to similar or identical images during training, potentially inflating performance through memorization rather than true generalization. Representative radiographic images were not included in the main manuscript due to attribution and licensing considerations, which may limit visual interpretability of findings.

Second, the included cases represent confirmed diagnoses, which may bias the dataset toward more advanced or radiographically classic presentations of disease. These cases are likely easier to detect compared to early or atypical tumors encountered in real-world clinical practice, where subtle findings and diagnostic uncertainty are more common. As a result, model performance in this study may overestimate real-world diagnostic capability.

Third, model performance was evaluated using static images and standardized prompts, which do not reflect real-world clinical workflows. In practice, radiographic interpretation incorporates clinical history, advanced imaging, and multidisciplinary input, all of which were not available to the models. This study did not include domain-specific medical imaging models (e.g., MedCLIP, CheXagent), which may demonstrate improved performance due to specialized training. However, many of these models are not publicly accessible or require technical implementation, limiting their applicability to real-world clinical and public use. Future studies directly comparing general-purpose multimodal models with domain-specific medical AI systems would be valuable. Future work may explore agent-based or multi-component artificial intelligence systems that integrate specialized modules for image analysis and clinical reasoning, which may improve diagnostic accuracy and reduce hallucination rates.

Finally, sample sizes within individual tumor categories were relatively small, limiting statistical power for tumor-specific comparisons. Larger, multi-institutional datasets would be necessary to validate these findings and improve generalizability. The dataset included radiographs obtained from multiple projections (e.g., anteroposterior and lateral views) across different anatomical locations and tumor types. However, projection-specific variability was not analyzed. Given the heterogeneity of tumor types, anatomical sites, and the limited number of images within individual projection subgroups, such analysis would be underpowered and potentially confounded. Future studies should evaluate projection-specific performance using larger, more standardized datasets.

## 6. Conclusions

Large language models demonstrated strong ability to identify radiographic abnormalities and detect the presence of bone tumors, with Gemini consistently showing the highest overall performance. However, this did not translate to accurate tumor classification, as all models showed limitations in distinguishing specific tumor subtypes. Performance was highest in morphologically distinct or benign lesions, such as osteochondroma and non-ossifying fibroma, and consistently poor in more challenging tumors such as Ewing sarcoma. While inclusion of the correct diagnosis within the differential improved performance, these findings suggest that current models are better suited for recognizing pathology than for making definitive diagnoses.

A key contribution of this study is the identification of a mismatch between confidence and accuracy, particularly in GPT-4.1, where incorrect predictions were frequently associated with high confidence. This finding is especially relevant given the increasing public accessibility of these tools, as non-specialists may use AI platforms to interpret radiographs outside of clinical settings. In this context, the combination of strong detection ability and high-confidence outputs may lead users to overinterpret findings as definitive diagnoses, increasing the risk of inappropriate reassurance or unnecessary alarm. Taken together, these results highlight the need for improved diagnostic specificity and better calibration of confidence before these tools can be safely integrated into clinical or public-facing use.

## Figures and Tables

**Table 1:** Distribution of radiographs and cases by diagnostic category included in the study dataset. A total of 257 radiographs representing 141 unique cases were analyzed, including normal studies and a spectrum of benign and malignant bone tumors.

Diagnosis	Images (*n*)	Cases (*n*)
**Normal**	81	44
**Osteosarcoma**	30	18
**Ewing Sarcoma**	29	18
**Enchondroma**	25	11
**Osteochondroma**	26	14
**Non-Ossifying Fibroma**	26	13
**Chondrosarcoma**	25	15
**Simple Bone Cyst**	15	8
**Total**	257	141

**Table 2:** Main. Overall diagnostic performance of large language models in radiograph interpretation. Metrics are reported as weighted percentages across all diagnostic categories. Abnormality detection and tumor detection were analyzed as separate outcomes. False positive abnormality rates represent normal radiographs incorrectly classified as abnormal, while tumor hallucination refers to incorrect identification of tumor in normal images. False negative rate represents the proportion of tumor-containing radiographs incorrectly classified as non-neoplastic and was calculated as the complement of tumor detection rate. Mean confidence reflects the weighted average confidence % of the AI modality across all cases. Confidence (Tumor Present) and Confidence (No Tumor) represent mean confidence stratified by tumor detection status. Pairwise comparisons between models were performed using chi-square testing. Statistically significant differences were observed across abnormality detection, tumor detection, and primary diagnostic accuracy, with Gemini demonstrating significantly higher detection rates, while GPT-4.1 showed significantly lower diagnostic accuracy compared to other models.

Model	Abnormality Detection (%)	Tumor Detection (%)	False Negative (%)	Primary Diagnostic Accuracy (%)	False Positive Abnormality (%)	Tumor Hallucination (%)	Mean Confidence (%)	Confidence (Tumor Present) (%)	Confidence (No Tumor) (%)
**ChatGPT 5.3**	88.7	75.2	24.8	52.6	13.5	0.0	85.4	83.5	91.5
**X-ray Interpreter 4.1**	82.4	70.1	29.9	36.2	40.7	0.0	91.1	84.0	96.0
**X-ray Interpreter Gemini**	97.8	93.4	6.6	56.1	25.9	12.3	88.6	87.5	92.5

**Table 4:** Tumor-specific diagnostic performance of large language models. Values are reported as percentages for each tumor category, with the number of radiographs (*n*) indicated. Tumor detection represents the proportion of cases correctly identified as neoplastic. Primary diagnostic accuracy reflects correct identification of the tumor subtype as the first (most likely) diagnosis. Conditional accuracy represents diagnostic accuracy among cases in which a tumor was correctly detected. Differential inclusion (top three) represents the proportion of cases in which the correct diagnosis was included within the first, second, or third ranked diagnoses. Performance varied substantially by tumor type, with higher accuracy observed in benign or morphologically distinct lesions and consistently poor classification of Ewing sarcoma across all models.

Tumor Type (*n* Images)	Model	Tumor Detection (%)	Primary Accuracy (%)	Conditional Accuracy (%)	Differential Inclusion (Top 3) (%)
**Osteosarcoma (** ***n*** ** = 30)**	ChatGPT 5.3	90.0	56.7	63.0	66.7
**Osteosarcoma (** ***n*** ** = 30)**	X-ray Interpreter 4.1	90.0	80.0	88.8	80.0
**Osteosarcoma (** ***n*** ** = 30)**	X-ray Interpreter Gemini	100.0	76.7	76.7	76.7
**Ewing Sarcoma (** ***n*** ** = 29)**	ChatGPT 5.3	31.0	0.0	0.0	0.0
**Ewing Sarcoma (** ***n*** ** = 29)**	X-ray Interpreter 4.1	58.6	0.0	0.0	27.6
**Ewing Sarcoma (** ***n*** ** = 29)**	X-ray Interpreter Gemini	96.6	10.3	10.7	37.9
**Enchondroma (** ***n*** ** = 25)**	ChatGPT 5.3	68.0	56.0	82.0	60.0
**Enchondroma (** ***n*** ** = 25)**	X-ray Interpreter 4.1	64.0	28.0	43.8	28.0
**Enchondroma (** ***n*** ** = 25)**	X-ray Interpreter Gemini	96.0	56.0	58.3	56.0
**Osteochondroma (** ***n*** ** = 26)**	ChatGPT 5.3	92.3	84.6	91.7	84.6
**Osteochondroma (** ***n*** ** = 26)**	X-ray Interpreter 4.1	65.4	23.1	35.3	27.0
**Osteochondroma (** ***n*** ** = 26)**	X-ray Interpreter Gemini	96.2	65.4	68.0	65.4
**Non-Ossifying Fibroma (** ***n*** ** = 26)**	ChatGPT 5.3	92.3	76.9	83.3	80.7
**Non-Ossifying Fibroma (** ***n*** ** = 26)**	X-ray Interpreter 4.1	80.8	42.3	52.4	52.6
**Non-Ossifying Fibroma (** ***n*** ** = 26)**	X-ray Interpreter Gemini	100.0	76.9	76.9	96.1
**Simple Bone Cyst (** ***n*** ** = 15)**	ChatGPT 5.3	60.0	33.3	44.4	80.0
**Simple Bone Cyst (** ***n*** ** = 15)**	X-ray Interpreter 4.1	53.3	20.0	37.5	26.7
**Simple Bone Cyst (** ***n*** ** = 15)**	X-ray Interpreter Gemini	100.0	33.3	33.3	93.3
**Chondrosarcoma (** ***n*** ** = 25)**	ChatGPT 5.3	100.0	20.0	20.0	52.0
**Chondrosarcoma (** ***n*** ** = 25)**	X-ray Interpreter 4.1	60.0	4.0	6.6	24.0
**Chondrosarcoma (** ***n*** ** = 25)**	X-ray Interpreter Gemini	84.0	32.0	38.1	56.0

## Data Availability

The datasets generated during the current study are available from the corresponding author upon request.

## Supplementary Materials

The following supporting information can be downloaded at: https://www.mdpi.com/article/10.3390/diagnostics16101460/s1, Table S1: Comprehensive list of Radiopaedia cases used in this study, including tumor category, case identifiers (rID), contributing authors, and source URLs for each radiograph.
